# Light Plays an Essential Role in Intracellular Distribution of Auxin Efflux Carrier PIN2 in *Arabidopsis thaliana*

**DOI:** 10.1371/journal.pone.0001510

**Published:** 2008-01-30

**Authors:** Ashverya Laxmi, Jianwei Pan, Mustafa Morsy, Rujin Chen

**Affiliations:** Plant Biology Division, Samuel Roberts Noble Foundation, Ardmore, Oklahoma, United States of America; Temasek Life Sciences Laboratory, Singapore

## Abstract

**Background:**

Light plays a key role in multiple plant developmental processes. It has been shown that root development is modulated by shoot-localized light signaling and requires shoot-derived transport of the plant hormone, auxin. However, the mechanism by which light regulates root development is not largely understood. In plants, the endogenous auxin, indole-3-acetic acid, is directionally transported by plasma-membrane (PM)-localized auxin influx and efflux carriers in transporting cells. Remarkably, the auxin efflux carrier PIN proteins exhibit asymmetric PM localization, determining the polarity of auxin transport. Similar to PM-resident receptors and transporters in animal and yeast cells, PIN proteins undergo constitutive cycling between the PM and endosomal compartments. Auxin plays multiple roles in PIN protein intracellular trafficking, inhibiting PIN2 endocytosis at some concentrations and promoting PIN2 degradation at others. However, how PIN proteins are turned over in plant cells is yet to be addressed.

**Methodology and Principle Findings:**

Using laser confocal scanning microscopy, and physiological and molecular genetic approaches, here, we show that in dark-grown seedlings, the PM localization of auxin efflux carrier PIN2 was largely reduced, and, in addition, PIN2 signal was detected in vacuolar compartments. This is in contrast to light-grown seedlings where PIN2 was predominantly PM-localized. In light-grown plants after shift to dark or to continuous red or far-red light, PIN2 also accumulated in vacuolar compartments. We show that PIN2 vacuolar targeting was derived from the PM via endocytic trafficking and inhibited by HY5-dependent light signaling. In addition, the ubiquitin 26S proteasome is involved in the process, since its inhibition by mutations in *COP9* and a proteasome inhibitor MG132 impaired the process.

**Conclusions and Significance:**

Collectively, our data indicate that light plays an essential role in PIN2 intracellular trafficking, promoting PM-localization in the presence of light and, on the other hand, vacuolar targeting for protein degradation in the absence of light. Based on these results, we postulate that light regulation of root development is mediated at least in part by changes in the intracellular distribution of auxin efflux carriers, PIN proteins, in response to the light environment.

## Introduction

As a prominent environmental signal, light plays an essential role in plant developmental processes including seed germination, seedling de-etiolation, leaf expansion, stem elongation, phototropism and flowering [1], [2]. When germinated in the absence of light, plants develop long hypocotyls with unexpanded cotyledons and exaggerated apical hooks. In contrast, when grown in the presence of light, plants develop short hypocotyls and fully expanded cotyledons. This differential growth in response to light is essential for plants to survive in the natural environment, allowing plants to quickly emerge from soil after germination to initiate photoautotrophic growth. In a shaded environment, it allows plants to elongate to compete for a limited light source [3].

Light signals are perceived by sensory photoreceptor proteins including phytochromes for red and far-red lights, cryptochromes for blue and UV-A light, phototropins and an uncharacterized photoreceptor for UV-B light [4]. In Arabidopsis, five phytochromes (PHYA-PHYE) have been identified, among which PHYA and PHYB are two major photoreceptors for far-red and red light, respectively. Light signaling involves nucleo-cytoplasmic partitioning of phytochromes [5] and negative regulators such as CONSTITUTIVE PHOTOMORPHOGENIC 1 (COP1), an E3 ubiquitin ligase involved in 26S proteasome-mediated protein degradation [6], [7]. In darkness, COP1 accumulates in the nucleus to degrade transcription factors including HY5, HYH, HFR and LAF, therefore, suppressing the expression of light-regulated genes [8]–[13]. On the other hand, light triggers the degradation of COP1 in the nucleus, thereby, activating the expression of light-regulated genes, and promoting photomorphogenesis. The cytoplasmic-nuclear partitioning of COP1 is regulated by the multisubunit COP9 complex (COP9 signalosome or CSN). Loss-of-function mutations in any CSN components exclude COP1 from nuclear accumulation in dark [14].

Recent studies suggest that shoot-localized phytochromes regulate lateral root development [15], and root-localized phytochromes and cryptochromes regulate phototropic responses and growth of primary roots [16]–[19]. Although, it has been shown that light regulates developmental processes at least in part through cross-talk with the phytohormone auxin [20], [21], the mechanism by which light and auxin interact to regulate root development is largely unknown. In plants, indole-3-acetic acid (IAA), a major endogenous auxin is polarly transported by a specific transport system that includes AUX1 and PIN families of auxin influx and efflux carrier proteins [22]–[32]. PIN proteins exhibit characteristic asymmetric localization in the plasma-membrane (PM) of auxin-transporting cells, determining the direction of auxin flow [33], [34]. In Arabidopsis, at least five *PIN* genes are expressed in different or overlapping groups of cells in roots, maintaining an auxin sink and maximum in the root tip [35], [36], generating a lateral auxin gradient in root cap columella cells upon gravi-stimulation [37], and controlling basipetal auxin transport in root distal and elongation zones [38]–[42]. PIN proteins undergo constitutive recycling between the PM and endosomes [43]–[46]. This intracellular trafficking process is rather dynamic and directly modulated by auxin and environmental factors such as light and gravity [47]–[49]. Remarkably, recent studies suggest that the endocytosis of PIN proteins is highly specified. PIN1 used a GNOM (GDP/GTP Exchange Factor for ARF GTPase) endosomal pathway [43], [44], while PIN2 used a different pathway involving SNX1 (sorting nexin) for intracellular trafficking [45], [46]. It has been shown that endocytosis is closely linked to degradation of PIN2 in root gravitropic response [49]. However, our understanding underlying intracellular trafficking and turnover of PIN proteins is still largely limited.

Here we show that light plays an important role in the maintenance of the PM-localization of the auxin efflux regulator, PIN2, in root epidermal and cortical cells. In the absence of light, the steady state level of PIN2 on the PM was greatly reduced, and PIN2 location in part switched from the PM to vacuoles. We show that the vacuolar PIN2 was derived from the PM via endocytosis. We further investigated the mechanism by which PIN2 undergoes vacuolar internalization, using monochromatic light treatments, and pharmacological inhibitors and mutants that affect photomorphogenesis and ubiquitin 26S proteasome.

## Results

### Auxin efflux regulator PIN proteins undergo vacuolar accumulation in dark-grown roots

**Figure 1:**
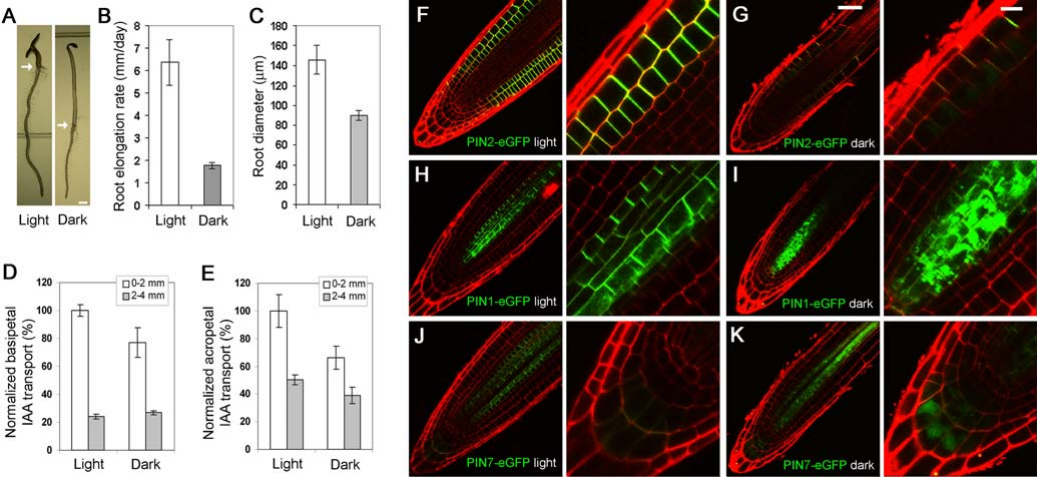
Root growth, auxin transport and intracellular localization of PIN proteins in Arabidopsis plants grown in the presence and absence of light. (A) A 5-day-old seedling grown under light developed a long root, short hypocotyl and two fully-expanded cotyledons (left); by contrast, a dark-grown seedling developed a short root, long hypocotyl, two un-expanded cotyledons and an apical hook (right). Arrows marked hypocotyl-root junction. (B) Root elongation rate was 6.4±1 and 1.8±0.1 mm/day for light- and dark-grown plants, respectively (n = 10; repeated three times, *p*<0.05). (C) Root diameter was 148±12 and 90±5 µm for light- and dark-grown seedlings, respectively (n = 10; repeated three times, *p*<0.05). Normalized root basipetal auxin transport (D) and acropetal auxin transport (E) in dark-grown plants was 77% and 50% that of light-grown counterparts (n = 8; repeated three times, *p*<0.05). (F-K) Shown were median optical sections of root tips of plants grown in light (F, H, I) and dark (G, I, K), expressing PIN2-eGFP (F, G), PIN1-eGFP (H, I) and PIN7-eGFP (J, K), and counter stained for cell walls with propidium iodide (red). Error bars represent standard deviations. Scale bars, 2 mm (A); 50 µm (F-K; left panels); 10 µm (F-K; right panels).

Compared to that of plants grown in continuous light, root radial expansion and rate of root elongation of plants grown in dark were reduced by 38% (90±5 vs. 148±12 µm) and 72% (1.8±0.1 vs. 6.4±1 mm/day), respectively (Figs. 1A–C). In addition, root gravitropic response was greatly reduced in plants grown in dark compared to the light-grown counterparts (data not shown). The phenotypes of dark-grown plants mimics that of auxin transport mutants [36], suggesting that auxin transport may be affected in plants grown in dark. To test this, we measured auxin transport activities in roots of 5-day-old plants grown either in continuous light or in dark. Because radial expansion was significantly reduced in the root tip region of dark-grown plants, which may indirectly affect auxin transport measurements, auxin transport activities were normalized, taken into account the differences in root radial expansion. The normalized data show that root acropetal (base-to-tip) and basipetal (tip-to-base) auxin transport activities in dark-grown plants were significantly reduced to 50% and 77%, respectively, that of light-grown plants (Figs. 1D, E; n = 8, three replicates, *t*-test *p*<0.05). The reduced root basipetal auxin transport in dark-grown plants was similar to that of light-grown *agr1–5* mutant (an allele of *pin2* mutants; [50].

To elucidate mechanism by which auxin transport was significantly reduced in dark-grown roots, we examined the localization pattern of PIN2 protein, a key regulator of root basipetal auxin transport [38]–[42]. Laser confocal scanning microscopic (LCSM) analysis of a functional PIN2-eGFP fusion protein driven by the native PIN2 promoter indicated that the PIN2-eGFP fusion protein was properly localized to the apical end of root epidermis cells, consistent with previous reports [36], [51]. In root cortical cells, however, PIN2-eGFP exhibited two opposite polarities, i.e. to the apical end of cells in the proximal region and to the basal end of cells in the distal region [29], [40]. The latter pattern of PIN2 localization is required to maintain the auxin maximum in the root tip [52], [53] and PINOID protein kinase and PP2A phosphatase have been shown to play a role in PIN2 polarity in root cortical cells [54].

**Figure 2:**
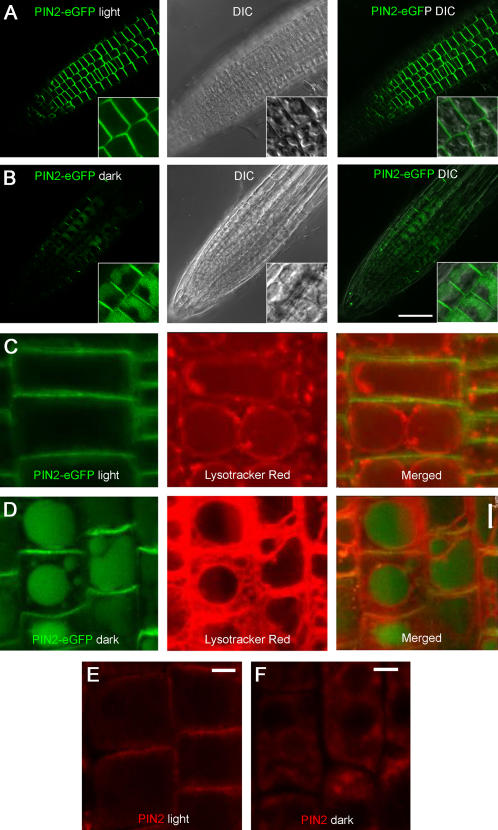
PIN2-eGFP vacuolar accumulation in dark-grown plants. (A) PIN2-eGFP (green) asymmetric localization at the apical plasmamembrane (PM) of root epidermis cells of 5-day-old light-grown plants (left; inset). No vacuolar accumulation of PIN2-eGFP was observed by DIC imaging (middle and right; insets). (B) In dark-grown seedlings, PIN2-eGFP PM-localization was greatly reduced (left; inset) and a detectable level of PIN2-eGFP accumulated in vacuolar compartments (middle and right; insets). (C, D) Lysotracker red (red) labeling of vacuolar compartments in root epidermis cells of light-grown (C; middle) and dark-grown (D; middle) plants. PIN2-eGFP only accumulated in vacuoles of dark-grown plants (C, D). Insets were close-up images. (E, F) Immuno-fluorescence labeling of the endogenous PIN2 protein in light-grown and dark-grown wild type plants. The endogenous PIN2 protein was localized to the apical end of root epidermal cells of a 5-day-old light-grown plant (E; red). In 5-day-old dark-grown plants, PIN2 was greatly reduced from the PM, and localized in vacuolar compartments (F; red). Scale bar, 50 µm (A, B), 10 µm (C–F).

By contrast, in 5-day-old seedlings grown in dark, PIN2-eGFP was greatly reduced from the PM and a detectable level of PIN2-eGFP was accumulated in intracellular compartments resembling vacuoles in both epidermal and cortical cells (Figs. 1F, G; 2A, B; 4A, B). To confirm that PIN2-eGFP intracellular compartments were vacuoles, we carried out differential interference contrast (DIC) microscopic analysis of root epidermis cells (Figs. 2A, B; middle panels) and labeling experiments with lysotracker red, a fluorescence dye that specifically marks acidic endomembrane compartments (Figs. 2C, D; middle panels). These data collectively confirmed that the intracellular compartments where PIN2-eGFP accumulated in dark-grown, but not in light-grown plants, were vacuolar compartments. Immuno-fluorescence labeling of the endogenous PIN2 proteins, using affinity-purified anti-PIN2 antibodies [50], confirms that the PIN2-eGFP fluorescence patterns in light- and dark-grown seedlings represent the patterns of the endogenous protein (Figs. 2E, F).

**Figure 3:**
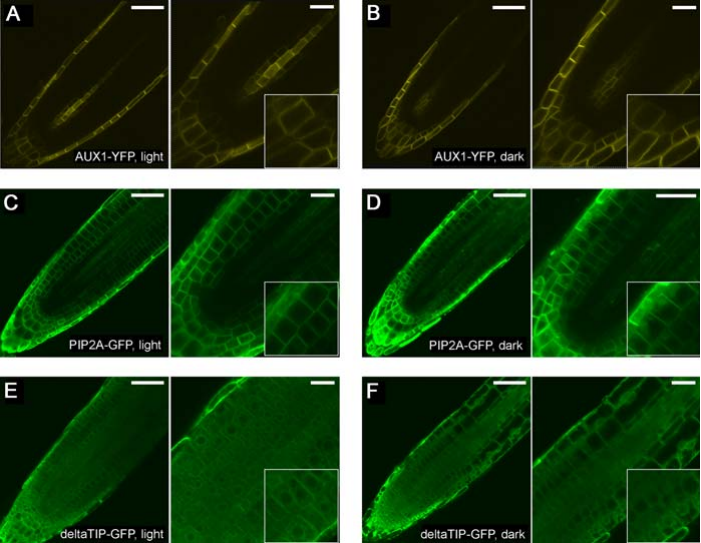
Localization of AUX1-YFP, PIP2A-GFP and deltaTIP-GFP in light- and dark-grown plants. AUX1-YFP was mainly localized to the basal PM of lateral root cap and central pre-vascular cells, and to the PM of root columella cells in both light-grown (A) and dark-grown (B) seedling roots. PIP2A-GFP was predominantly on the PM of all root cells, except that it was excluded from the quiescent center and surrounding initial cells, of light-grown plants (C). In dark-grown seedlings, a detectable level of PIP2A-GFP accumulated in vacuolar compartments (D). deltaTIP-GFP labeled both the PM and tonoplast membrane of root cells of light-grown (E) and dark-grown (F) plants. Shown in right and insets were close-up images. Scale bars, 50 µm (left), 25 µm (right).

To determine whether the vacuolar accumulation of PIN2 occurred via a specific or general process, we examined localization patterns of several other PM-resident proteins. We observed that several other PIN proteins including PIN1 [32], [55] and PIN7 [36] similarly accumulated in vacuoles of several different types of root cells of dark-grown seedlings, where PIN proteins were expressed (Figs. 1H–K). Furthermore, a PM-localized water channel PIP2A [56] also changed from predominant PM-location in light-grown seedlings to both PM and vacuolar locations in dark-grown seedlings (Figs. 3C, D; insets). Surprisingly, the presumptive auxin influx carrier AUX1 did not significantly alter its intracellular localization in three different types of cells, root columella, lateral root cap and central pre-vascular cells of dark-grown seedlings (Figs. 3A, B; insets). The vacuolar structure, as indicated by the pattern of a deltaTIP-GFP fusion protein that marks both the PM and tonoplast membrane [56], [57], remained largely unchanged in the meristematic region of the root grown in dark compared to the light-grown counterpart (Figs. 3E, F; insets). Taken together, our data suggest that PIN2 vacuolar accumulation in roots of dark-grown seedlings takes place via a process that is shared by a subset of PM-resident proteins.

**Figure 4:**
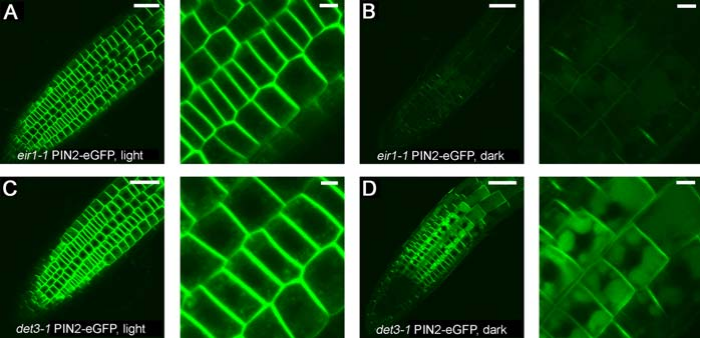
Enhanced PIN2-eGFP vacuolar accumulation in dark-grown *det3-1* mutant. PIN2-eGFP was slightly enhanced in diffuse and punctate cytoplasmic structures in light-grown *det3-1* mutant, compared to the *eir1-1* control plant (A, C). In dark-grown *det3-1* mutant, a high level of PIN2-eGFP was detected both at the PM and in vacuolar compartments, compared to the dark-grown control plant, where PIN2-eGFP was greatly reduced from the PM and a greatly reduced level accumulated in vacuolar compartments (B, D). Shown were root epidermal cells imaged under identical confocal settings. Scale bars, 50 µm (left), 10 µm (right).

Previously, vacuolar-targeted GFP was observed in vacuoles of dark-grown plants, but not in light-grown plants [58], [59]. This phenomenon was attributed to an impaired vacuolar function, resulting in GFP accumulation in vacuoles of only dark-grown plants [58]. In light-grown seedlings, however, vacuolar fluorescence was minimized due to rapid degradation of the green fluorescence protein entering the vacuolar lumen [58], [59]. Because PIN proteins do not appear to contain any recognizable vacuolar targeting sequences, it is not clear whether PIN2-eGFP vacuolar accumulation in dark-grown plants was simply due to an impaired vacuolar function as previously reported for vacuolar-targeted GFP. Alternatively, the dark-treatment may alter intracellular distribution of PIN2, promoting sorting from late endosomes to vacuoles and subsequent accumulation in vacuolar lumen due to reduced vacuolar function. To distinguish these possibilities, we tested intracellular PIN2-eGFP localization in a homozygous *det3-1* mutant, in which the vacuolar H^+^-ATPase activity was impaired, resulting in reduced vacuolar function [60]. We found that in the light-grown *det3-1* mutant, although a slightly elevated level of PIN2-eGFP was detected in diffuse and punctate structures in the cytoplasm compared to the wild type plants, PIN2-eGFP was mainly restricted to the PM (Figs. 4A, C). By contrast, in the dark-grown *det3-1* plants, strong PIN2-eGFP signals were detected both in the vacuolar compartments and at the PM (Fig. 4D). These patterns were drastically different from the weak PIN2-eGFP at the PM and in the vacuoles of the wild type plants grown in dark (Fig. 4B), suggesting that protein degradation in the vacuolar compartments still took place so that PIN2-eGFP did not accumulate to a high level in the vacuoles of dark-grown wild type plants. Our data collectively support the alternative hypothesis that, unlike vacuolar-targeted GFP, PIN2-eGFP vacuolar accumulation was likely caused by a combination of increased vacuolar targeting and reduced vacuolar function in dark-grown wild type plants. However, our data do not rule out the possibility that vacuolar targeting of PIN2-eGFP at a reduced magnitude may also take place in light-grown plants.

### PIN2 undergoes vacuolar accumulation in light-grown seedlings after shift to dark via PM-derived endosomes

**Figure 5:**
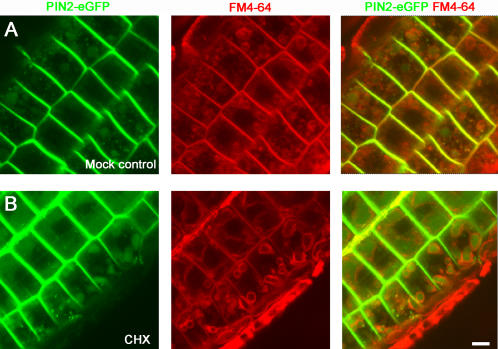
Vacuolar accumulation of PIN2-eGFP did not require de novo protein synthesis. Five-day-old light-grown PIN2-eGFP seedlings were pulse-labeled with an endocytosis marker, FM4-64, and pre-treated on growth media with or without cycloheximide (CHX; 50 µM) for 30 min. The plants were then shifted to dark and incubated for 4 hrs. In the absence of cycloheximide, PIN2-eGFP (green) accumulated in vacuolar compartments marked by FM4-64 (red) (A). Similarly, in the presence of cycloheximide, PIN2-eGFP also accumulated in vacuolar compartments in plants after shift to dark (B). Shown were root epidermis cells. Scale bar, 10 µm.

**Figure 6:**
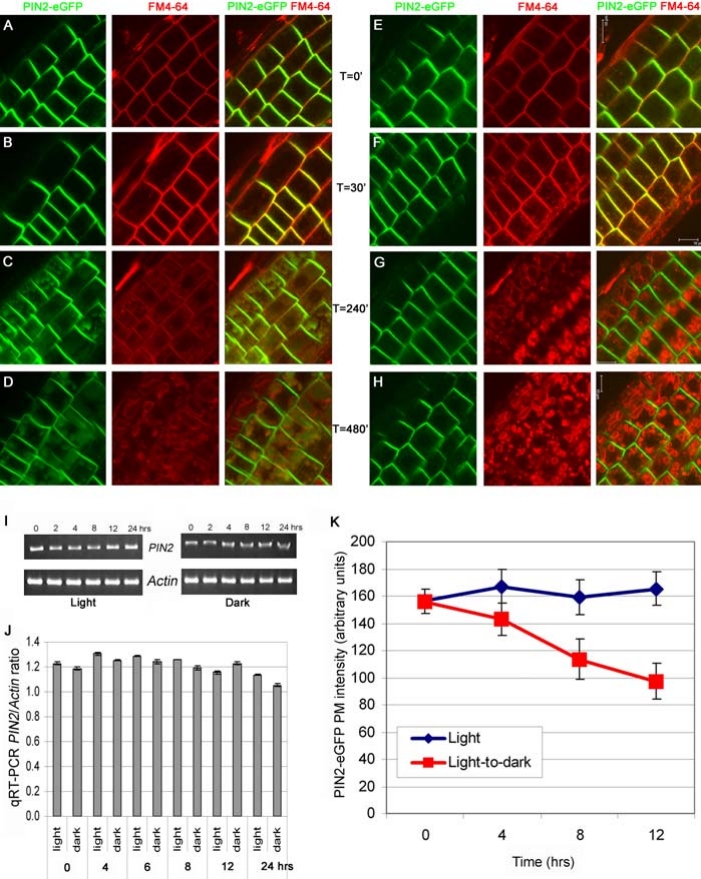
Time course of PIN2-eGFP vacuolar targeting. Five-day-old light-grown PIN2-eGFP seedlings were pulse-labeled with an endocytosis marker, FM4-64, and then transferred to dark (A–D) or kept in light (E–H). Both PIN2-eGFP (green) and FM4-64 (red) were restricted to the plasma membrane (PM) at T = 0 (A, E). At T = 30 min after transfer to dark, FM4-64 internalized to early endosomes and marked PIN2-eGFP-labeled endosomes (B). At T = 4 hrs after transfer to dark, PIN2-eGFP accumulated in vacuolar compartments, whose membrane was now labeled with FM4-64 (C). At T = 8 hrs after transfer to dark, strong vacuolar accumulation of PIN2-eGFP and FM4-64 labeling of vacuolar membrane were visible (D). On the other hand, FM4-64 labeled endosomes at T = 30 min (F) and vacuolar compartments at T = 4 and 8 hrs in seedling kept in the light condition (E–H). But, PIN2-eGFP remained at the PM under light (E–H). (A–H) Shown were root epidermal cells. Left, PIN2-eGFP (green); middle, FM4-64 (red); right, merged images. Scale bars, 10 µm. (I) RT-PCR analysis of steady state *PIN2* transcript levels in seedlings grown in continuous light (left) or after light-to-dark transition (right) for up to 24 hrs. The steady state transcript levels of an *Actin* gene were used as internal loading controls. (J) Real-time qRT-PCR analysis of steady state *PIN2* levels, normalized against the level of the *Actin* gene. Shown were the average *PIN2/Actin* ratios of three independent experiments. (K) Fluorescence intensities of PIN2-eGFP at the PM of root epidermis cells of light-grown plants kept in light (blue line) or shifted to dark (red line) for 0, 4, 8 and 12 hrs. Significant differences were observed between light- and dark-shifted plants at T = 4, 8 and 12 hrs (n = 168–466; Student's *t*-test, *p*<0.0001).

It has been previously shown that PIN2 undergoes constitutive cycling between the PM and endosomal compartments [45], [46], [61], [62]. However, vacuolar targeting of PIN2 protein has not been described so far. To gain insights of the mechanism underlying PIN2 intracellular distribution, we tested whether PIN2 accumulates to vacuolar compartments in light-grown seedlings after a short-term shift to dark. We found that PIN2-eGFP accumulated in vacuolar compartments in light-grown plants after shift to dark for a short period time (Figs. 5A; 6A–D).

Next, we tested whether PIN2 vacuolar accumulation during the light-to-dark transition requires de novo protein synthesis. We observed that in the presence of a protein synthesis inhibitor, cycloheximide, PIN2-eGFP accumulated in vacuolar compartments in light-grown seedlings after shift to dark for 4 hrs (Fig. 5B). Although our data do not rule out the possibility that some of the vacuolar PIN2-eGFP may be derived from the biosynthetic trafficking from the endoplasmic reticulum (ER), these data, together with the labeling of the vacuolar membrane with an endocytosis marker, FM4-64, strongly support that vacuolar PIN2-eGFP was largely derived from the endocytic trafficking from the PM (Figs. 5A, B).

To demonstrate that vacuolar PIN2-eGFP was derived from the PM via endocytic vesicle trafficking, we pulse-labeled 5-day-old light-grown PIN2-eGFP seedlings with an endocytic marker, FM4-64 and examined the kinetics of FM4-64 endocytosis and PIN2-eGFP vacuolar accumulation in a time course after shifting seedlings to dark. We observed that both FM4-64 (red) and PIN2-eGFP (green) fluorescent signals were restricted to the PM at T = 0 (Fig. 6A). However, within 15–30 min in dark, FM4-64 was internalized and labeled early endosomes (Fig. 6B). We observed that PIN2-eGFP-positive endosomes partially overlapped with FM4-64-labeled endosomes (Fig. 6B), in agreement with previous observations that PIN2-eGFP undergoes constitutive cycling between the PM and early endosomes [61], and that different groups of endosomes are involved in the endocytosis of different PIN proteins in Arabidopsis roots [45], [61]. Within 4 to 8 hrs after transfer to dark, PIN2-eGFP accumulated in vacuolar compartments, whose membrane was now marked by FM4-64 (Figs. 6C, D). In seedlings grown in continuous light, the kinetics of FM4-64 uptake was indistinguishable from that of the light-to-dark transitioned seedlings (Figs. 6E–H). However, in contrast to that of dark-shifted plants, no detectable level of PIN2-eGFP accumulated in vacuoles of the light-grown seedlings (Figs. 6E-H). Taken together, our data strongly support that the fraction of PIN2-eGFP accumulated in vacuoles after the light-to-dark shift was derived from the PM via endosomal vesicles. Furthermore, our data suggest that the general endocytic trafficking as indicated by the uptake of the endocytic marker, FM4-64, was not significantly altered by the light-to-dark shift (Figs. 6A–H).

Internalization into lytic compartments is often associated with turnover of PM-resident proteins of both animals and plants [63], [64]. To test whether this is the case, we first examined PIN2 steady state transcript level after light-to-dark transition, using semi-quantitative and quantitative real-time RT-PCR. These data indicate that PIN2 steady state transcript level normalized against the internal *Actin* gene fluctuated within a small range (5–7%) in light-to-dark-shifted plants in comparison with plants kept in continuous light during a 24-hr time course (Figs. 6I, J), suggesting that transcriptional regulation may not play a significant role in the intracellular distribution of PIN2-eGFP in response to the light condition. We then quantified fluorescence intensities of PIN2-eGFP on the PM of root epidermal cells from 9–11 individual plants, using a semi-quantitative confocal microscopy [47]. The results indicate that PIN2-eGFP PM signal remained little changed in plants kept in the continuous light condition (Fig. 6K; blue line; n = 168–330; 9–11 individual plants; two independent experiments; *t*-test, *p*>0.5). However, in plants shifted to dark, PIN2-eGFP signal was gradually reduced over time, reaching 62% of the initial level in 12 hrs (Fig. 6K; red line; n = 168–466; 9–11 individual plants; two independent experiments; *t*-test, *p*<0.0001).

### Light signaling is required for the PM-localization and vacuolar targeting of PIN2

**Figure 7:**
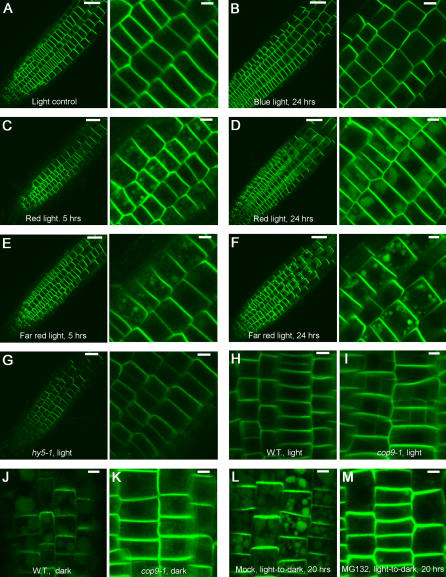
Light signaling in the regulation of PIN2 intracellular distribution. (A) PIN2-eGFP was localized at the apical PM of root epidermal cells of plants grown in continuous white light. (B) PIN2-eGFP asymmetric location in root epidermis cells was maintained in light-grown plants shifted to continuous blue light (475 nm) for 24 hrs. When light-grown plants were shifted to continuous red light (660 nm) for 5 hrs (C) or 24 hrs (D), PIN2-eGFP accumulated in vacuolar compartments. When light-grown plants were shifted to continuous far red light (730 nm) for 5 hrs (E) or 24 hrs (F), PIN2-eGFP accumulated in vacuolar compartments. (G) PIN2-eGFP was greatly reduced from the PM of root epidermis cells of light-grown *hy5-1* mutant. PIN2-eGFP was moderately enhanced at the PM in light-grown homozygous *cop9-1* mutant (I), compared to that of the wild type plant (H). In dark-grown *cop9-1* mutant, PIN2-eGFP PM localization was enhanced and vacuolar accumulation was reduced (K), compared to that of the dark-grown wild type plant (J). In the presence of MG132 (50 µM), PIN2-eGFP PM localization was enhanced, while vacuolar accumulation was reduced in the light-grown plant after shift to dark for 20 hrs (M), compared to the reduced PM localization and increased vacuolar accumulation in the wild type plant after shift to dark for 20 hrs (L). Shown in right (A–G) were close-up images. Scale bars, 50 µm (A–G, left); 10 µm (A–G, right; H–M).

To examine whether PIN2 vacuolar targeting depends on specific light signaling or results from physiological changes in plants grown in light and dark, we tested whether photoreceptor-dependent light signaling is involved, using monochromatic light treatments. For this, we transferred 5-d-old seedlings grown in continuous white light to continuous blue (475 nm), red (660 nm) or far-red (730 nm) light for various lengths of time, and examined the intracellular distribution of PIN2-eGFP. We observed that, when white light-grown seedlings were shifted to continuous blue light (475 nm) for various time, PIN2-eGFP remained at the PM, similar to that of light-grown seedlings (Fig. 7A, B). Thus, these observations indicate that blue light was sufficient to maintain the steady state PIN2 PM-location. On the other hand, when white light-grown seedlings were transferred to continuous red (660 nm) or far red (730 nm) light for 4 to 5 hrs, a detectable level of PIN2-eGFP already accumulated in vacuolar compartments (Figs. 7C, E), similar to that of light-grown plants after shift to dark. In a prolonged incubation in continuous red and far red light, a significant level of PIN2-eGFP accumulated in vacuolar compartments (Figs. 7D, F). These data indicate that phytochrome-dependent red/far red light signaling was not sufficient to maintain PIN2-eGFP PM-localization.

**Figure 8:**
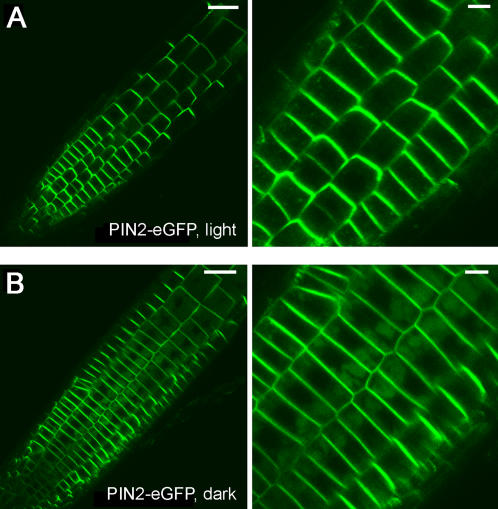
PIN2-eGFP intracellular distribution in excised roots. Four-day-old light grown PIN2-eGFP seedlings, after removal of cotyledons, were transferred to either light (A) or dark (B) for 10 hrs. PIN2-eGFP was localized to the PM in excised roots incubated in the presence of light (A). By contrast, PIN2-eGFP was accumulated in vacuolar compartments in excised roots incubated in the absence of light (B). Shown in right were close-up images. Scale bars, 50 µm (left); 10 µm (right).

We tested whether similar patterns of PIN2 intracellular distribution occur in excised roots in response to the light environment. For this, we removed cotyledons from 4-day-old light-grown seedlings and transferred the excised roots to either continuous light or dark for 10 hrs, and examined the PIN2-eGFP localization. These data indicate that PIN2-eGFP was accumulated in vacuolar compartments of excised roots incubated in absence of light, but not of excised roots incubated in the presence of light (Figs. 8A, B). These data suggest that excised roots similar to intact whole seedlings can sense the light condition in regulating PIN2 protein intracellular localization.

Because light signaling requires HY5-dependent transcriptional regulation of light responsive genes, we reasoned that, if HY5-dependent downstream events are involved in the regulation of PIN2 PM-localization, we would expect to see reduced PIN2 PM-localization in light-grown *hy5* mutants, similar to that of wild type plants grown in dark. Furthermore, if this is the case, we would also expect to see increased PIN2 PM-localization in dark-grown *cop9* mutants, in which light-responsive genes are expressed. As expected, we observed that PIN2-eGFP PM-localization was greatly reduced in light-grown *hy5-1* mutant plants compared to the light-grown wild type plants (Figs. 7A, G). On the other hand, in dark-grown *cop9-1* mutants, PIN2-eGFP PM-localization was significantly enhanced and PIN2-eGFP vacuolar accumulation was reduced compared with the wild type plants grown in dark (Figs. 7J, K). In light-grown *cop9-1* roots, PIN2-eGFP PM-localization was only moderately enhanced compared with the light-grown wild type plants (Figs. 7H, I). Collectively, these data support a model that HY5-dependent light signaling plays an important role in PIN2 intracellular distribution, maintaining its PM-location and inhibiting vacuolar targeting.

Because COP9 signalosome is directly involved in the modulation of the ubiquitin E3 ligase function, which in turn catalyzes ubiquitination of substrates and marks them for 26S proteasome-mediated degradation, and, recently, the 26S proteasome has been implicated in the auxin-regulated PIN2 protein degradation during gravitropism [49], we tested whether the 26S proteasome also plays a role in PIN2-eGFP intracellular distribution in response to the light condition. We observed that in the presence of MG132, an inhibitor of the 26S proteasome, PIN2-eGFP vacuolar accumulation was greatly reduced and PM-localization was enhanced in light-grown seedlings after transfer to dark for an extended time, in contrast to the mock-treated control plants (Figs. 7L, M). The effect of MG132 was quite similar to that of the *cop9-1* mutation, suggestive of a role for the 26S proteasome in PIN2 intracellular distribution.

## Discussions

In this study, we show that light plays an important role in the regulation of intracellular distribution of auxin efflux regulator PIN2 protein, maintaining its PM localization and reducing vacuolar targeting for protein turnover. Evidence that supports a role of light signaling in the intracellular distribution of PIN2 protein includes (1) shifting light-grown plants to continuous blue light did not alter the intracellular distribution of PIN2-eGFP; (2) shifting light-grown plants to continuous red and far red light did, however, favor PIN2-eGFP vacuolar accumulation; (3) in dark-grown photomorphogenetic mutant, *cop9-1*, we observed a high level PM-localization and reduced vacuolar accumulation of PIN2-eGFP; (5) in light-grown *hy5* mutant defective in light-regulated gene expression and in root growth and gravitropic response [65], PIN2-eGFP PM-localization was greatly reduced; and (6) in the presence of the ubiquitin 26S proteasome inhibitor, MG132, PIN2-eGFP PM-localization was maintained and vacuolar accumulation was reduced in light-grown plants after shift to dark for an extended time.

In animal and yeast cells, endocytosis and subsequent degradation of PM proteins and receptors in lysosomes/vacuoles are used to effectively down-regulate signaling processes associated with receptors and transport activities of transporters. Endocytosis and degradation of the yeast general amino acid permease Gal1, the zinc transporter Zrt1, the magnesium transporter Alr1, the sugar transporters Mal1, Mal6, Gal2, and Hxt6/7, and the uracil transporter Fur4 are all promoted when the substrate levels are elevated [66]. Thus, regulating the activity of transport proteins via endocytic trafficking is a critical process for organisms to respond to changing nutrient availability. In plant cells, the boron exporter BOR1 accumulates to a high level under conditions of B limitation, mediating B translocation from roots to shoots. However, when B supply is high, BOR1 undergoes endocytosis and vacuolar degradation [64]. On the other hand, the Arabidopsis BKI1 (BRI1 Kinase Inhibitor 1), a membrane-bound repressor of the brassinosteroid receptor kinase BRI1, is rapidly endocytosed upon binding with brassinolide [67]. Interestingly, the auxin efflux carrier PIN2 also undergoes endocytosis and proteolysis in cells of the upper flank of gravi-stimulated roots [49]. Even though auxin has been implicated in this process [49], the role of auxin in PIN2 endocytosis and degradation is not fully understood, since auxin also inhibits PIN2 endocytosis in cells of the bottom flank of gravi-stimulated roots [47]. It is possible that the diverse function of auxin may be explained by dosage effects and/or other factors that are likely involved. So far, vacuolar targeting and degradation of PIN2 has not been reported.

AGR1/EIR1/PIN2 is a key regulator of root basipetal auxin transport and gravitropic response [38]-[41]. Similar to PIN1, another member of the PIN family of auxin efflux carriers, PIN2 protein undergoes constitutive cycling between the PM and endosomal compartments in root epidermal and cortical cells [36], [61]. In this study, we show that PIN2, together with several other PM-resident proteins including PIN1, PIN7 and PIP2A, but not AUX1, the presumptive auxin influx carrier, is targeted to vacuolar compartments in the absence of light. Visualization of PIN2-eGFP vacuolar targeting was in part due to reduced vacuolar function in plants grown in dark. This is consistent with previous observations that vacuolar targeted green fluorescence protein accumulated in vacuolar lumens of only dark-grown plants. We observed that in dark-grown *det3-1* plants that are defective in vacuolar function due to an impaired H^+^-ATPase activity, PIN2-eGFP accumulates to a high level both at the PM and in vacuoles compared to the dark-grown wild type plants. Our data do not rule out the possibility that PIN2-eGFP may be targeted to vacuoles in the presence of light. However, this process is apparently not favored in plants grown in light.

At present, it is not clear what the immediate regulator is for PIN2 protein vacuolar targeting in the absence of light. Because PIN2 can be phosphorylated and ubiquitinated [49], [54], it is tempting to speculate that phosphorylation or ubiquination, or both, are likely involved. Auxin itself may be an alternative regulator of PIN2 endocytosis and vacuolar targeting in the absence of light, since the shoot-derived auxin transport is also affected in dark-grown plants (Fig. 1E; [15]). The kinetics of PIN2 endocytosis and vacuolar targeting in dark was relatively slow compared to the rapid endocytosis of BKI1 induced by brassinolide [67], but, similar to the B-induced turn-over of BOR1 transporter [64]. This is suggestive of involvement of intermediate processes that are likely responsive to the nutrient availability in case of BOR1 and to the diurnal fluctuation of the light environment in case of PIN2.

Auxin transport also plays an important role in hypocotyl elongation in light-grown but not in dark-grown Arabidopsis plants [20]. Furthermore, Arabidopsis hypocotyls were impaired in the negative gravitropic response in the presence of continuous red light [19]. These observations are in agreement with our data that other PIN proteins are likely involved in these physiological processes [15], [37]. On the other hand, the *p*-glycoproteins, PGP1 and MDR1/PGP11, have been shown to regulate hypocotyl elongation in a phytochrome-dependent manner [68], [69]. PGP1 and MDR1/PGP11 play a role in auxin transport in hypocotyls [70], [71]. It is imperative to test whether *p*-glycoproteins are also subject to the light regulation at the level of intracellular trafficking. Interestingly, exogenous application of cytokinin and ethylene restored the negative hypocotyl gravitropic response in the presence of red light, suggestive of cross-talk between light signaling and hormonal pathways [19], [72], [73]. Future work in elucidating the mechanism by which light regulates intracellular trafficking of auxin efflux carrier proteins promises to shed new insight in our understanding of interactions between light and signaling pathways that impinge on plant developmental processes.

## Materials and Methods

### Materials and Growth Conditions

All seed stocks were obtained from the Arabidopsis Biological Resource Center at Ohio State University except that *PIN1::PIN1-eGFP* (L*er* background), *PIN2::PIN2-eGFP* (*eir1-1* background), *PIN7::PIN7-eGFP* (Col-0 background) and *AUX1::AUX1-YFP* (Col-0 background) lines were kindly provided by Jeffrey Long, Ben Scheres, Jiri Friml and Malcolm Bennett, respectively. To generate *cop9-1 PIN2-eGFP* lines, a *cop9-1* heterozygote (Ws; ♀) was crossed with PIN2-eGFP (*eir1-1*; ♂). F_1_ plants were self-pollinated and grown to F_2_. 30 F_2_ lines positive for PIN2-eGFP were self-pollinated and grown to F_3_. Several F_3_ lines segregating for the *cop9-1* mutation and homozygous for PIN2-eGFP were selected. Homozygous *cop9-1* lines and wild type siblings were used in the study. Similarly, to generate *hy5-1 PIN2-eGFP* and *det3-1 PIN2-eGFP* lines, homozygous *hy5-1* (L*er-*0*; ♀*) and *det3-1* (Col-0; *♀)* was crossed with *PIN2-eGFP* (*eir1-1*; ♂). Homozygous F3 lines were selected based on long hypocotyl phenotype of the *hy5-1* mutant in the presence of light, short hypocotyl phenotype of *det3-1* in dark, and PIN2-eGFP. F3 homozygous mutant lines and wild type siblings were used in the study.

Seeds were surface sterilized essentially as described previously (Shin et al., 2005). After imbibition at 4°C for 1–2 days, seeds were germinated and grown vertically on Petri dishes containing 0.5× Murashige and Skoog basal salts with minimal organic medium (MSMO; Sigma) supplemented with 1% sucrose and solidified with 0.8% Type E Agar (Sigma). Seed germination was carried out in climate-controlled growth rooms in a long day condition (16hr light and 8hr darkness), except stated otherwise, with 22/20°C day/night temperature and 80 µmol/sec/m^2^ light intensity. For growth in monochromatic light, light sources from Norlux Monochromatic Hex (NHX) solid-state light modules (NorLux Corp) were used. The intensity of red (660 nm), far-red (730 nm), and blue (475 nm) light was 10, 4, and 10 µmol/m^2^/s, respectively.

All chemicals were from Sigma except specified otherwise, and prepared as stock solutions. DMSO was used to dissolve MG132 (25 mM) and lysotracker red (2 mM; Invitrogen). Propidium iodide (10 mM; Invitrogen) and *N*-(3-triethylammoniumpropyl)-4-(6-(4-(diethylamino) phenyl) hexatrienyl) pyridinium dibromide (FM4-64; 2.5 mM; Molecular Probes) were dissolved in water. 1-NAA (10 mM) and IAA (10 mM) were first dissolved in 1N NaOH and then diluted with water.

### Labeling with FM4-64, lysotracker red and propidium iodide

For uptake studies of FM4-64, 4–5 day-old light-grown seedlings were first incubated in water containing 2.5 µM FM4-64 for 5 minutes. The seedlings were then transferred back to the growth medium plate and incubated in either continuous light or darkness for various length of time before being subjected to laser confocal scanning microscopic observation. For labeling with lysotracker red, 4–5 day-old seedlings grown either under continuous light or in darkness were incubated in water supplemented with lysotracker red (2 µM final concentration) for 1h before laser confocal imaging analysis. For labeling of cell wall with propidium iodide, seedlings were incubated in propidium iodide solution (10 µM) for 30 seconds before confocal imaging analysis.

### RT-PCR and real-time qRT-PCR

Six-day-old wild type (Col-0) seedlings grown in a long day condition (16 hrs of light and 8 hrs of dark) were either kept in light or transferred to dark by wrapping the plates with aluminum foil. Seedlings were collected and immediately frozen in liquid nitrogen at the following time points, 0, 2, 4, 8, 12 and 24 hrs after the treatments. Three biological replicates were collected for each time point. Total RNA was isolated using RNeasy Plant Mini Kit (Qiagen). Residual DNA contaminants were removed by treating RNA samples with RNase-free DNases (20 units). One microgram of total RNA was used to synthesize the first strand cDNAs with SuperScript III cDNA Synthesis Kit (Invitrogen). The cDNA templates were then used in PCR amplification in 20 or 24 cycles of *PIN2* and *Actin* (AT5G09810) transcripts with the following gene-specific primers: (PIN2-f) 5′ CCGTGGGGCTAAGCTTCTCATCT 3′; (PIN2-r) 5′ AGCTTTCCGTCGTCTCCTATCTCC 3′; (Actin-f) 5′ CAGTGTCTGGATCGGAGGAT 3′ and (Actin-r) 5′ TGAACAATCGATGGACCTGA 3′. PCR products were resolved by electrophoresis in 1.2% agarose-ethidium bromide gels. Gels were scanned by a Typhoon Trio scanner and data were analyzed by ImageQuant2.1 software (Amersham Biosciences). Expression of the constitutively expressed *Actin* gene was used as an internal control.

For real-time qRT-PCR, PCR reactions were performed in an optical 384-well plate with an ABI PRISM 7900 HT sequence detection system (Applied Biosystems, Foster City, CA, USA), using SYBR Green to monitor dsDNA synthesis. Reactions were performed in a 10 µl volume contained 5 µl 2× SYBR Green Master Mix (Applied Biosystems), 1.0 ng cDNA and 1 µm of each gene-specific primer. PCR cycles were performed as: 50°C for 2 min; 95°C for 10 min; 40 cycles of 95°C for 15 sec and 60°C for 1 min. Data was collected and analyzed using the SDS 2.2.1 software (Applied Biosystems). Primer titration and dissociation experiments were performed to ensure that no primer dimmers or false amplicons will interfere with the result. Following the real-time PCR experiment, C_T_ values for *PIN2* gene were normalized to the C_T_ value of the reference *Actin* gene.

### Laser Confocal Scanning Microscopy (LCSM)

GFP, FM4-64, lysotracker red, propidium iodide and rhodamine fluorescence was imaged under a Leica TCS SP2 AOBS Laser Confocal Scanning Microscope (Leica Microsystems, Exton, PA). For imaging GFP, the 488 nm line of the Argon laser was used for excitation and emission was detected at 520 nm. For imaging FM4-64, lysotracker red, propidium iodide and rhodamine, 543 nm line of the Helium/Neon laser was used for excitation and emission was detected at 590–620 nm. Differential interference contrast (DIC) images were captured using the transmission light detector of the confocal microscope. For semi-quantitative measurement of fluorescence intensities, laser, pinhole and gain settings of the confocal microscope were kept identical among treatments. Digital images were analyzed for fluorescence intensities using Metamorph 6 (Molecular Devices). Images were assembled using Photoshop version 5.0 (Adobe Systems).

### Immuno-fluorescence localization of PIN2 protein

Immuno-fluorescence labeling of PIN2 was carried out essentially as described previously [50]. Primary polyclonal antibodies were raised in rabbits and affinity purified as described before [50]. They were used as 1∶200 dilutions. Secondary antibodies (rhodamine-conjugated goat anti-rabbit IgG antibodies; Jackson Lab) were used as 1∶300 dilutions. After washing with a saline solution for three times 20 min each, the samples were inspected, using LCSM.

### Auxin transport assay

Root basipetal auxin transport was measured essentially as previously described (Shin et al, 2005). Root acropetal auxin transport was carried out as described in Buer and Muday (2004) with modifications. Briefly, agar blocks of 1 mm in diameter containing 7.7×10^−8 ^M ^3^H-IAA (Amersham) was applied at the hypocotyl-root junction. After incubation for 5 hrs, a 0.5 mm section of the root close to the agar block was dissected and discarded. Two consecutive 2-mm segments below the incision line were then collected separately and pooled from 6 to 10 roots and placed into glass scintillation vials containing 5 mL scintillation fluid. Radio-activities in these two pools of root segments were measured using a Beckman Coulter LS6500 Scintillation counter (Fullerton, CA, USA). The amount of the radioactivity was the average of three separate experiments±standard deviation. Student's *t*-test with paired two-tailed distribution was used for statistical analysis.
